# Topical Application of Synthetic Hormones Terminated Reproductive Diapause of a Univoltine Weed Biological Control Agent

**DOI:** 10.3390/insects12090834

**Published:** 2021-09-16

**Authors:** Ikju Park, Lincoln Smith

**Affiliations:** 1 Invasive Species and Pollinator Health Research Unit, USDA ARS Western Regional Research Center, 800 Buchanan St., Albany, CA 94706, USA; link.smith@usda.gov; 2 Department of Entomology and Nematology, University of California Davis, Davis, CA 95616, USA; 3 School of Applied Bioscience, Kyungpook National University, Daegu 41566, Korea

**Keywords:** 20-hydroxyecdysone (20E), methoprene, *Ceratapion basicorne*, *Centaurea solstitialis*, yellow starthistle

## Abstract

**Simple Summary:**

Yellow starthistle (*Centaurea solstitialis*) is an invasive annual plant that has infested multi-million ha in the western United States, causing multi-billion dollars of losses and management costs. The rosette weevil (*Ceratapion basicorne*) has recently been approved for biological control of this weed. However, this weevil reproduces only once a year, which hinders mass-rearing for release. Here, we tested whether insect hormones can break the reproductive diapause of female weevils. We found that applying two insect hormones, 20E and methoprene, can consistently terminate diapause of female weevils to enable them to lay eggs. Thus, topically applying insect hormones could be used to induce females to lay eggs at any time of year, which would permit rearing more than one generation per year. This could greatly increase the number of weevils produced per year in a mass-rearing program to accelerate the release of rosette weevils to help suppress yellow starthistle in the United States. These insects would revert to having one generation per year after release in synchrony with their host plant.

**Abstract:**

Classical biological control is an important method for controlling invasive alien weeds. Univoltine insects can be highly effective biological control agents of annual weeds because they are well synchronized with their host plant. However, having only one generation per year makes it difficult and slow to multiply them in the laboratory for initial field releases. If it were possible to terminate reproductive diapause early, then we could rear multiple generations per year, which would greatly increase annual production. We used a recently approved biocontrol agent, *Ceratapion basicorne* (a univoltine weevil), for yellow starthistle (*Centaurea solstitialis*) as a model system to study the use of two insect hormones, 20-hydroxyecdysone (20E) and methoprene, to terminate reproductive diapause. Methoprene (1 μg applied topically) terminated reproductive diapause of female weevils, whereas doses of 0.0, 0.01 and 0.1 μg did not. The combination of methoprene and 20E had a stronger effect and induced an increase in eggs (1.51 ± 0.16 eggs/day, mean ± SE) compared with a methoprene only group (1.00 ± 0.13 eggs/day), and a control group (0.21 ± 0.04 eggs/day). Thus, topical application of these hormones should enable us to rear the weevil out of its normal season and produce more than one generation per year, which will increase productivity of mass-rearing it for field release. Once released in the field, the insect would continue as a univoltine agent that is well-synchronized with its host plant.

## 1. Introduction

Classical biological control, the use of host-specific natural enemies, is an important strategy for controlling invasive alien plants [1]. Once new classical biocontrol agents are approved for field release, they must be multiplied and released so that they can become widely established in order to successfully control the target weed. Furthermore, the initial number of biocontrol agents released at a site is critical for their establishment [2,3], so it is important to produce as many agents as possible. Multivoltine insects are relatively easy to multiply, but many biological control agents, particularly of annual target weeds, are univoltine. Such univoltine species typically have obligatory diapause, which enables them to be synchronized with their host plant. However, this makes it more difficult to multiply them because normally only one generation can be produced in a year. If it were possible to terminate such diapause, then it would be possible to produce more generations in laboratory rearing, which could increase the annual production of agents to release in the field. Beetles, especially weevils, have often been used for biological control of weeds [4], and many of them experience reproductive diapause in the adult stage. Furthermore, diapause has been identified as one of the principal causes of rearing failure in weed biological control programs [5]. Thus, it is important to understand the factors that induce and terminate reproductive diapause of biological control agents [6,7]. The phases of diapause include: induction, preparation, initiation, maintenance, termination and post-diapause quiescence [8]. Species that have an obligatory hereditary stage-specific arrest of development (parapause) pose the greatest challenge to manipulation [9]. Termination of diapause can be controlled by a combination of physiological and environmental changes. A variety of environmental conditions that are associated with seasonal changes are known to terminate reproductive diapause of adult insects, and the specific requirements have been studied in a number of species, especially agronomic pests [7].

Yellow starthistle (*Centaurea solstitialis* L., Asteraceae) is an invasive alien annual weed that has infested about 6 million ha in the Western United States [10]. It costs about USD 1.4 billion in California alone [11]. Six biocontrol agents were released that attack the flower heads [12,13], as well as a rust fungus that attacks the leaves and stems [14]. However, there is still a need for another biocontrol agent that attacks the rosette stage of yellow starthistle [15]. *Ceratapion basicorne* Illiger (Coleoptera: Apionidae) is a univoltine weevil that feeds and develops on the rosette of yellow starthistle that was approved for release in 2019 [16,17,18,19]. Adult weevils emerge from winter diapause in the early spring and feed and oviposit on rosette leaves, and larvae tunnel down the leaf petioles and develop in the root crown, where they pupate [20,21]. Adult progeny emerge in late May to June in Italy, and they feed and mate for a few weeks then disappear to aestivate and hibernate until the following spring. Females in diapause feed much less than ovipositing females (a mean of 4.1 vs. 19.2 holes per day), and feeding rate is highly correlated to oviposition rate [21]. Methods have been developed to rear the weevil on potted plants, but the obligatory reproductive diapause prevents producing more than one generation per year. The underlying mechanism regulating reproductive diapause of *C. basicorne* has not been previously studied. However, if we could discover methods to terminate the reproductive diapause, then we would be able to rear multiple generations of *C. basicorne* in the laboratory, which would greatly increase the numbers for release.

Reproductive diapause of insects is generally regulated by two hormones: juvenile hormone (JH) secreted from the corpora allata (CA) and ecdysone secreted from both the prothoracic gland in larval stages [7,22] and reproductive organs including the ovaries of females and accessary glands of males [23,24]. Reduced JH titer induces reproductive diapause [25,26], which is caused by either JH esterase activity [27,28] or blocking the JH biosynthesis pathway [29]. Further, 20-hydroxyecdysone (20E), which is the active form of ecdysone, is necessary for JH production and terminating reproductive diapause [23]. Methoprene, which is the closest structurally to natural JH among synthetic JH analogs, has been used as an insecticide in the insect growth regulator class [30]. It prevents molting to kill juvenile insects, but it also promotes vitellogenesis in adult insects [30]. JH treatment is able to induce the oogenesis of female Colorado potato beetles (*Leptinotarsa decemlineata* [Say], Coleoptera: Chrysomelidae), but only temporarily, and 20E alone did not terminate reproductive diapause [31]. However, application of 20E followed by JH permanently terminated the reproductive diapause of Colorado potato beetle in the laboratory [31]. Similarly, the combined application of 20E and methoprene had a greater effect on termination of reproductive diapause of female face flies (*Musca autumnalis* De Geer, Diptera: Muscidae) than treatment of either hormone alone [26]. Termination of reproductive diapause by regulating the abovementioned hormones has been demonstrated in the following insect orders: Coleoptera [31,32,33,34], Diptera [35], Hemiptera [36], Hymenoptera [37] and Lepidoptera [38,39,40]. However, to the best of our knowledge, the idea of using synthetic hormones to terminate reproductive diapause has not been examined regarding helping to rear biocontrol agents for weeds.

The purpose of this study is to measure the effect of two synthetic hormone analogs to terminate the reproductive diapause of female *C. basicorne*, which would enable them to start laying fertile eggs and facilitate rearing weevils in the laboratory for the field release. We measured the effect of (1) different concentrations of methoprene and (2) preceding the methoprene treatment with 20E to stimulate an increase in adult feeding and oviposition of *C. basicorne*.

## 2. Materials and Methods

### 2.1. Insect Rearing and Plant Material

Adult *C. basicorne* emerged from potted yellow starthistle plants in the greenhouse at ambient photoperiod and temperature from 02/03/2020 to 04/03/2020. They were placed in transparent plastic cups with organdy screen tops, and provided cut yellow starthistle leaves inserted in sealed water vials for food. 

Adults were observed mating when held at room temperature. Weevils were held in a refrigerator (Whirlpool, Benton Harbor, MI, USA) at 5 °C under 24 h darkness, and were removed for a few hours one day a week to allow them to feed and to minimize cold stress [41], until further use. During these warming periods, males copulated with females even though the latter may have still been in reproductive diapause (not able to oviposit). Tests were conducted at the following conditions (LD 12:12, 21 °C: 17 °C) and 60–80% (RH) in an incubator (Percival, Perry, IA, USA), which simulates spring conditions at which reproduction has previously been observed [20].

### 2.2. Methoprene and 20-Hydroxyecdysone

Methoprene was purchased from Sigma Aldrich (33375-100MG, Sulpelco, St. Louis, MO, USA). Methoprene was dissolved in acetone (≥99.9%, 650601-1L, Sigma Aldrich, St. Louis, MO, USA) to make a stock solution under a fume hood. The stock solution was made by adding 0.892 mL of acetone to a vial containing 100 mg of methoprene, which is equivalent to 100 mg/mL. The stock solution was serially diluted to produce concentrations of 1.0, 0.1 and 0.01 μg/μL. Topical application of 1 μL of each solution was applied on the abdomen of female *C. basicorne* by two squirts from a 0.5 μL Hamilton microsyringe (22184-U, 86250, Hamilton, Reno, NV, USA) under a stereo-microscope. An equal volume of acetone (1 μL) was topically applied to the control group.

In addition, 20-Hydroxyecdysone was purchased (H5142-5MG, Sigma Aldrich, St. Louis, MO, USA). Instead of dissolving in methanol as used in other studies [42,43,44], we dissolved 20E in the acetone to be consistent with the methoprene experiment. Another study also showed that the topical application of 20E dissolved in acetone worked on the Mediterranean flour moth, *Ephestia kuehniella* Zeller (Lepidoptera: Pyralidae) [39]. Instead of direct injection or oral administration, we chose to use topical application for two reasons. Injection involves piercing the insect, which we thought would be very harmful, especially for a small hard-bodied weevil (abdomen < 2 mm long). Second, oral administration was not an appropriate method for this species, because adults feed inside the leaves, so it would not be possible to apply a known dose. Topical application of 20E using alcohols or acetone have previously been used successfully on other types of insects [45,46,47]. We used 1 μg of 20E because 20E (1 μg) and methoprene (1 μg) caused 92% of diapause-destined female face flies, *Musca autumnalis*, to become reproductively active [26]. We conducted four different experiments to test the relationship between methoprene and 20E to terminate the reproductive diapause of female *C. basicorne* in the laboratory.

#### 2.2.1. Experiment 1: Methoprene Dose–Response Experiment

Female weevils went through the following environmental conditions before conducting Experiment 1: (1) an overwinter condition at 5 °C:5 °C under a 0:24 h light:dark photoperiod for four weeks, (2) a late spring condition at 22 °C:17 °C and 12:12 h LD for a week and adjusted to 22 °C:17 °C and 15:09 h LD for three weeks, and (3) an aestivation condition at 30 °C:22 °C and 15:09 h LD for eight weeks. We conducted oviposition experiments during the late spring condition to the aestivation condition, but none of the females oviposited in yellow starthistle leaves. To identify the concentration of methoprene that terminates diapause of females, we used the following concentrations: 1.0, 0.1, 0.01 μg of methoprene dissolved in 1 μL of acetone, and a control (=1 μL of acetone only) on Day 0 (4 female weevils for control; 5 females each for 1.0, 0.1 and 0.01 μg of methoprene). Each female was placed in a container with a cut yellow starthistle leaf for food and oviposition site and held in an incubator (I-30BLL, Percival Scientific Inc., Perry, IA, USA) with four Philips 17-watt Alto II linear fluorescent tube light bulbs in the following environmental conditions: LD 12:12, 21 °C:17 °C and 60–80% RH. The leaf was replaced every day. We recorded the number of feeding holes and eggs for each female per day. Experiment 1 was conducted from 06/17/2020 to 07/01/2020.

#### 2.2.2. Experiment 2: Application of 1 μg Methoprene to Unresponsive Females

To evaluate whether 1 μg methoprene induces previously unresponsive females in Experiment 1 to lay eggs, we divided unresponsive females in Experiment 1 into two groups. The first group was topically treated with 1 μg methoprene dissolved in 1 μL acetone and the control group received only 1 μL acetone (*n* = 6 female weevils). “Unresponsive females” are defined as females that did not lay any eggs after one of the treatments used in Experiment 1. Because 1 μg methoprene made some females lay eggs in Experiment 1, females that previously received the 1 μg dose but did not lay eggs were excluded. Females in both groups were held with an excised leaf of the rosette stage of yellow starthistle that was replaced daily for two weeks. We recorded the number of feeding holes and eggs for each female per day. Because additional light sources increase the number of eggs in the mass-rearing process [48], we added an additional incandescent light bulb (75 watts, BR30, Philips, Somerset, NJ) with a 24-h mechanical timer inside the same incubator used in Experiment 1 (LD 12:12, 21 °C:17 °C). Experiment 2 was conducted from 07/16/2020 to 07/30/2020.

#### 2.2.3. Experiment 3: The effect of 20E on the Methoprene Treatment

To investigate the role of 20E applied two days before application of methoprene, we conducted three treatments: (1) acetone + acetone [AA: control], (2) acetone + methoprene [AM], and 20E + methoprene [2M]. A 20E alone application was not tested because of the small number of females available and because 20E alone failed to terminate reproductive diapause in Colorado potato beetles [31]. Females had been held in the cold conditions (LD 0:24, 5 °C) for 12 weeks, pulled out and oviposition was checked in the laboratory at ambient temperature and photoperiod from 1 April 2020 to 23 April 2020, but none of them laid eggs on rosette leaves of yellow starthistle. They were then placed back to the refrigerator (LD 0:24, 5 °C) for six months. Other newly emerged female weevils in April 2020 were placed in the refrigerator for approximately six months. Both groups of females were used in Experiment 3, and the environmental conditions were the same as Experiment 2. Eight weevils were tested for each treatment. The first application (1 μg of 20E/μL acetone or acetone only) was conducted on Day 0, followed by the second application (1 μg of methoprene/μL acetone or acetone only) on Day 2. A yellow starthistle leaf was replaced every two days for 24 days. We recorded the number of feeding holes and eggs for each female every two days. The hatching rate of eggs among three treatments was measured by dissecting cut leaves under the microscope 6–8 days after the day when the weevils were changed. We also compared the fecundity with that of non-diapausing females [21] to examine the effects of hormonal treatment on female *C. basicorne*. Experiment 3 was conducted from 24 October 2020 to 17 November 2020. After Experiment 3, each reproductively active female was placed on potted yellow starthistle plants every two days for them to oviposit to rear adult weevils as described in [21].

#### 2.2.4. Experiment 4: Application of 2M on Underperforming and Unresponsive Females

To test whether the 2M treatment can induce egg laying on underperforming and unresponsive females used in Experiment 3, we applied 20E (1 μg) followed in 2 days by methoprene (1 μg) to underperforming and unresponsive females using the same methods as in Experiment 3. We define “underperforming females” as female weevils that oviposited several days and stopped oviposition (=a short oviposition period) or that oviposited less than 1 egg per day more than two consecutive days. “Unresponsive females” are defined as females that did not lay any eggs after one of the treatments used in Experiment 3. A total of 18 weevils were used with the same environmental conditions as Experiment 3. We recorded the number of feeding holes and eggs in each female every two days. Experiment 4 was conducted from 19 November to 21 December 2020.

### 2.3. Statistical Analysis

Because the normality of residuals was significantly different on the Shapiro–Wilk test, we used the Kruskal–Wallis test as a non-parametric test to examine whether different methoprene concentrations affected the number of feeding holes and eggs in Experiment 1 and whether the absence/presence of 20E and/or methoprene altered the number of feeding holes and eggs in Experiment 3. The two-parameter Hill equation (where *a* is the inflection point and *n* is the Hill coefficient, which controls the curvature) was fit to the data using nonlinear regression.

feeding holes/maximum feeding holes = 1/(1 + (*𝑎*/dose)*^𝑛^*)

In addition, the paired Wilcoxon signed-rank test was used to test whether the number of both feeding holes and eggs was different after the preoviposition period among individual females used in experiments 1 vs. 2 (e.g., methoprene retreatment from Day 8 to 14) and experiments 3 vs. 4 (e.g., 20E plus methoprene retreatment from Day 8 to 24). The unpaired *T*-test was conducted to test whether feeding holes and eggs were different among treatments in Experiment 2. The Chi-square test was used to test whether proportions of ovipositing females, number of eggs, hatching rates, and adult mortality were different among three treatments.

## 3. Results

### 3.1. Experiment 1: Methoprene Dose–Response Experiment

**Figure 1:**
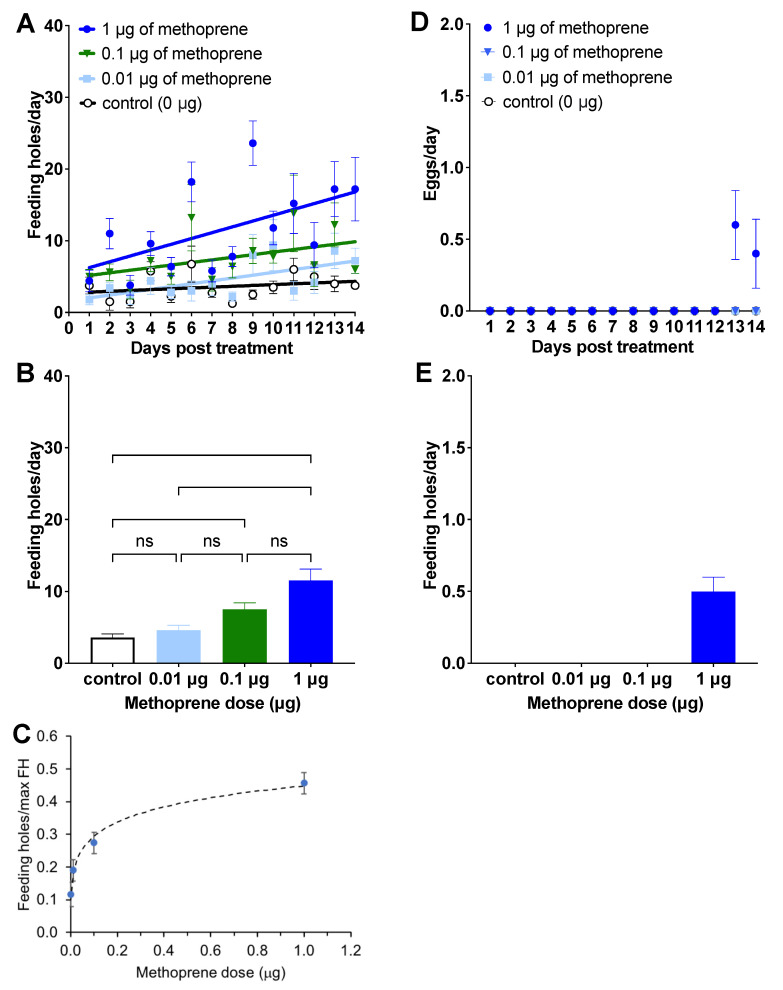
Effect of different doses of methoprene on feeding rate ((**A**)—feeding rate over time after treatment, (**B**)—mean feeding rate during 14 days; mean ± SE, (**C**)—the dose response curve) and oviposition ((**D**)—oviposition over time after treatment, (**E**)—mean oviposition during the oviposition period (Days 13–14); mean ± SE) of female *C. basicorne*. ns: not significant; *: *p* < 0.05; **: *p* < 0.01; ****: *p* < 0.0001. The solid lines in Figure 1A represent simple linear regressions.

Among the four treatments, 1 μg of methoprene tended to induce the highest number of feeding holes per day (1 μg: 11.53 ± 1.59, mean ± SE; 0.1 μg: 7.51 ± 0.89; 0.01 μg: 4.60 ± 0.69; 0 μg: 3.59 ± 0.47; Figure 1A,B). Both 1 μg and 0.1 μg of methoprene induced higher feeding than that of females treated in the control group (1.0 μg: *Z* = 4.404, *p* < 0.0001, 0.1 μg: *Z* = 3.100, *p* < 0.05, Figure 1B). There was no difference between 0.01 μg of methoprene and the control (*Z* = 0.840, *p* > 0.999) or between 0.01 μg and 0.1 μg of methoprene (*Z* = 2.260, *p* = 0.1429), or between 0.1 μg vs. 1 μg of methoprene (*Z* = 1.304, *p* > 0.999). However, the number of feeding holes was higher for females treated with 1 μg of methoprene than 0.01 μg of methoprene (*Z* = 3.564, *p* < 0.01). Linear regression analysis showed no increase in feeding rate over time for the control treatment (*F*_(1, 54)_ = 1.61, *p* = 0.21), but it did increase for the other three treatments (trt = 0.01: *F*_(1, 68)_ = 10.25, *p* = 0.0021, *Y* = 1.63 (±1.05 SE) + 0.396 (±0.123) * *X*; trt = 0.1: *F*_(1, 68)_ = 4.77, *p* = 0.032, *Y* = 4.80 (±1.41) + 0.361 (±0.166) * *X*; trt = 1.0: *F*_(1, 68)_ = 14.27, *p* > 0.0003, *Y* = 5.44 (±1.83) + 0.812 (±0.215) * *X,* Figure 1A). The dose–response curve using the Hill equation was fit to the ratio of feeding holes divided by the maximum number of feeding holes observed (which was 32), and is plotted in Figure 1C (fitted parameter estimates: *a* = 2.06, *n* = 0.290).

Egg-laying was observed only by females treated with 1 μg of methoprene, which occurred on Days 13 and 14 (Figure 1D). A total of 60% of females treated with 1 μg of methoprene oviposited on yellow starthistle leaves (a total of 5 eggs from 3 females) from Days 13 (0.60 ± 0.24; mean ± SE) to 14 (0.40 ± 0.24; Figure 1E). None of the weevils died during Experiment 1.

### 3.2. Experiment 2: Application of 1 μg Methoprene to Unresponsive Females

**Figure 2:**
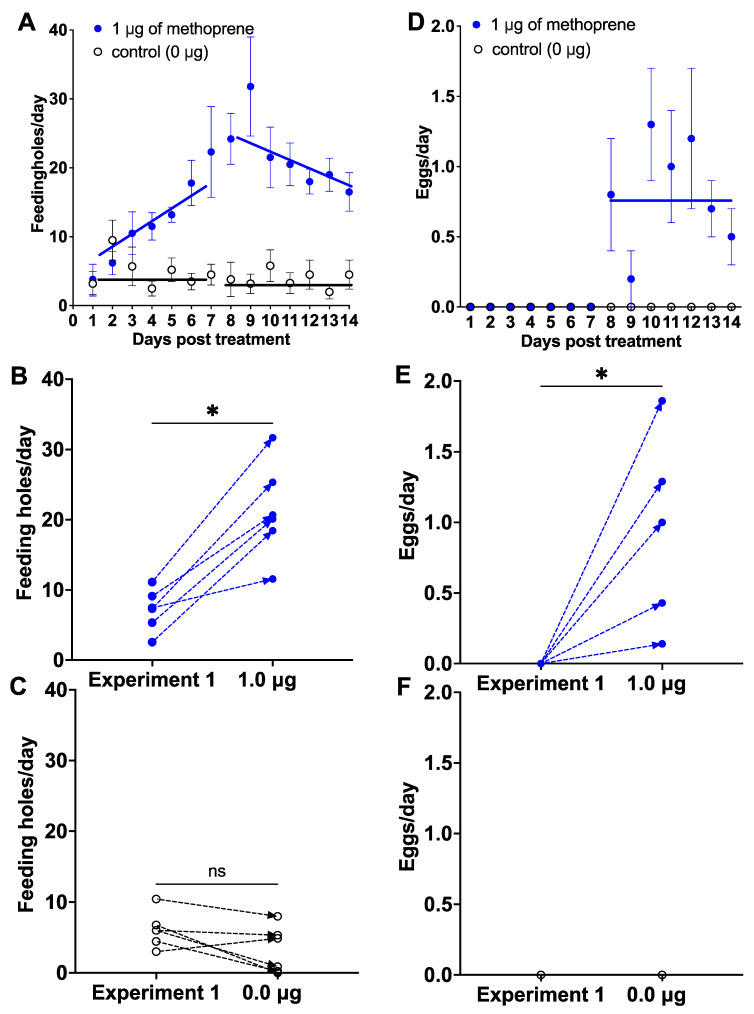
The effect of applying 1 μg of methoprene (blue dot) vs. control (1 μg of acetone; white dot) on females used in Experiment 1 that did not oviposit after being treated with 0, 0.1, or 0.01 μg of methoprene (Figure 1). Mean number ± SE of feeding holes over time ((**A**); solid lines represents linear regressions for the preoviposition and oviposition periods) and individual responses of each female treated with methoprene (**B**) and treated with acetone (**C**) in Experiment 1 and Experiment 2; mean number of eggs over time (**D**) and individual responses of each female retreated with methoprene (**E**) and with acetone (**F**) during the oviposition period; ns: not significant, *: *p* < 0.05, paired Wilcoxon signed-rank test.

Application of 1 μg of methoprene to females previously used in the control, 0.1 and 0.01 μg of methoprene treatments in Experiment 1 stimulated a gradual increase in the number of feeding holes from Day 1 to Day 9, which remained higher (21.64 ± 1.94; mean ± SE) than the feeding holes made by females in the control group from Day 8 to 14 (3.87 ± 0.46; unpaired *T*-test: *T* = 8.9, *p* < 0.0001, Figure 2A). Linear regression for the preoviposition period (Days 1–7) for the control was *Y* = 6.07 (±1.72 SE) − 0.304 (±0.384) * *X* (*F*_(1, 40)_ = 0.62, *p* = 0.43), and for 1.0 μg methoprene was *Y* = 0.55 (±2.64 SE) + 2.911 (±0.468) * *X* (*F*_(1, 40)_ = 24.30, *p* < 0.0001), and for the oviposition period (Day 8-14) for the control was *Y* = 4.54 (±3.90) − 0.060 (±0.348) * *X* (*F*_(1, 40)_ = 0.029, *p* = 0.87), and for 1.0 μg methoprene was *Y* = 42.14 (±8.27) − 1.863 (±0.740) * *X* (*F*_(1, 40)_ = 6.34, *p* = 0.016). The females ate more after treatment with 1 μg of methoprene than they did in Experiment 1, when treated with 0.0, 0.1 and 0.01 μg of methoprene (paired Wilcoxon signed-rank tests; *W* = 21.00, *p* < 0.05, Figure 2B) from Day 8 to 14, whereas the number of feeding holes was not different in the control group (*W* = −17.00, *p* = 0.0938, Figure 2C).

Application of 1 μg of methoprene induced females to oviposit from Day 8 to Day 14 (0.81 ± 0.15; mean ± SE), whereas none of the control females oviposited (unpaired *T*-test: *T* = 5.536, *p* < 0.0001; Figure 2D). Linear regression for the oviposition period (Day 8–14) was *Y* = 0.88 (±0.78) − 0.006 (±0.695) * *X* (*F*_(1, 40)_ = 0.0073, *p* < 0.93). Likewise, this was significantly higher for these same females than in Experiment 1 (0.0, 0.01, 0.1 μg), in which there was no oviposition (*W* = 21.00, *p* < 0.05, Figure 2E). A total of 100% of females treated with 1 μg of methoprene oviposited on yellow starthistle leaves vs. 0% for those treated with acetone only. A total of 28 eggs were collected from the 6 treated females from Day 8 to 14 and no eggs from the control group treated with acetone (Figure 2F). All female weevils treated with 1 μg methoprene were alive; however, 50% of female weevils in the control group were dead within 7 days.

### 3.3. Experiment 3: The Effect of 20E on the Methoprene Treatment

**Figure 3:**
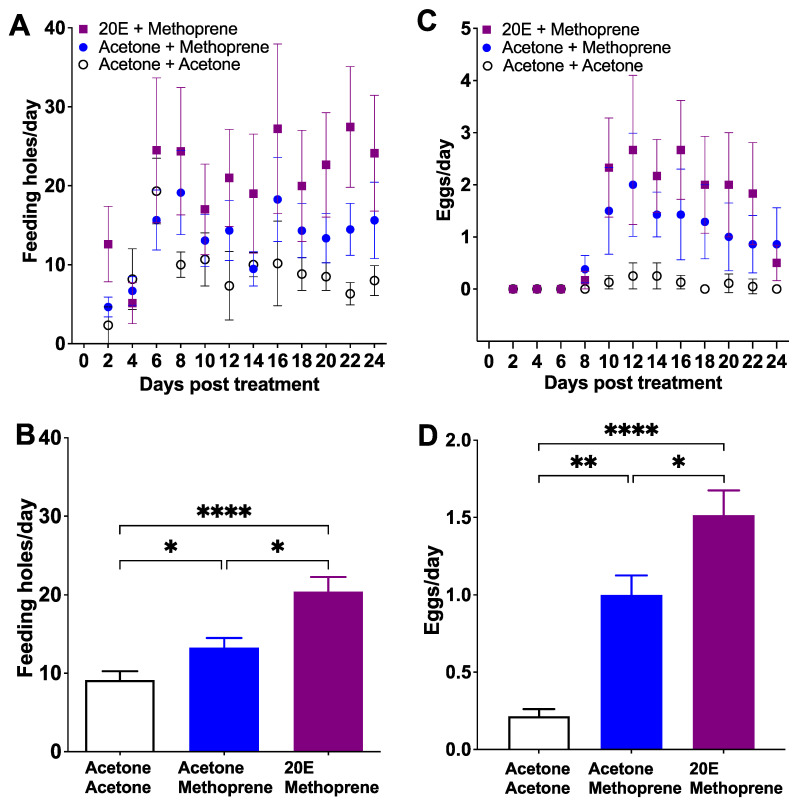
The effect of 20E and methoprene on feeding holes (**A**,**B**) and eggs (**C**,**D**) of female *C. basicorne* (mean ± SE). Day indicates the end of each two-day exposure period; acetone + acetone (white dot); acetone + methoprene (blue dot); 20E + methoprene (purple square); *: *p* < 0.05; **: *p* < 0.01; ****: *p* < 0.0001.

The 2M (20E + methoprene) treatment showed the highest number of feeding holes from Day 8 to 24 (22.54 ± 1.20 feeding holes/day, mean ± SE), followed by AM (acetone + methoprene; 14.68 ± 0.95 feeding holes/day) and AA (acetone + acetone; control; 8.87 ± 0.49 feeding holes/day) groups (*Z* = 21.20, *p* < 0.0001, Figure 3A). Like Experiments 1 and 2, females treated with 1 μg methoprene fed more than the control group (AM vs. AA, *Z* = −7.50, *p* < 0.05; Figure 3B). However, females treated with 20E and methoprene fed more than both the AM group (2M vs. AM, *Z* = −10.00, *p* < 0.05; Figure 3B) and the AA group (2M vs. AA, *Z* = 17.50, *p* < 0.0001; Figure 3B). Both 2M and AM groups started to lay eggs on Day 8, but on Day 10 for the AA group (Figure 3C). The egg production during the oviposition period (Day 8 to 24) in the 2M group (2.24 ± 0.13 eggs/day, mean ± SE) was higher than either the AM group (1.36 ± 0.04 eggs/day; *Z* = −8.708, *p* < 0.05; Figure 3D) or the AA group (0.13 ± 0.04 eggs/day; *Z* = −19.17, *p* < 0.0001; Figure 3D). Females in the AM group oviposited more eggs than the AA group (*Z* = −10.46, *p* < 0.05; Figure 3D). The proportion of females that oviposited was 83.3% for the 2M group, 62.5% for the AM group, and 37.5% from the AA group (χ^2^ = 48.87, df = 2, *p* < 0.0001). The proportion of eggs that were properly placed inside the leaf blade or midrib (the normal location that allows successful larval development) was 82.65%, 78.48% and 11.11% for the 2M, AM and AA groups, respectively (χ^2^ = 134.1, df = 2, *p* < 0.0001): 2M vs. AM (χ^2^ = 2.91, df = 1, *p* > 0.05), 2M vs. AA (χ^2^ = 42.37, df = 1, *p* < 0.0001), and AM vs. AA (χ^2^ = 24.80, df = 1, *p* < 0.0001). Eggs were also found on both the leaf surface (2M: 15.31%, AM: 20.25%, and AA: 88.89%) and midrib surface (2M: 2.04%, AM: 1.27%, and AA: 0%). Even if these eggs were fertile, they were not viable because they dessicated before embryos could complete development and eclose. Hatching rate of eggs that were placed inside leaves was 56.8%, 45.9% and 0% for the 2M, AM and AA groups, respectively (χ^2^ = 134.10, df = 2, *p* < 0.0001): 2M vs. AM (χ^2^ = 0.52, df = 1, *p* > 0.05), 2M vs. AA (χ^2^ = 104.10, df = 1, *p* < 0.0001), AM vs. AA (χ^2^ = 93.41, df = 1, *p* < 0.0001). The adult mortality was 25% for AA and 2M groups, and 12.5% for the AM group (χ^2^ = 5.787, df = 2, *p* > 0.05).

After Experiment 3, six female weevils treated with hormones (two females from the AM group and four females from the 2M group) oviposited viable eggs on potted plants for additional two months. The egg hatching rate was not explicitly examined on potted plants because it was difficult to check the 1st instar larvae inside the plant without damaging them. However, we reared out newly emerged weevils (*n* = 170) from the potted yellow starthistle plants. These adults were held at summer photoperiod for three months (at 19–24 °C) on cut yellow starthistle leaves, but did not oviposit, indicating that they were in reproductive diapause.

### 3.4. Experiment 4: Application of 2M on Underperforming and Unresponsive Females

**Figure 4:**
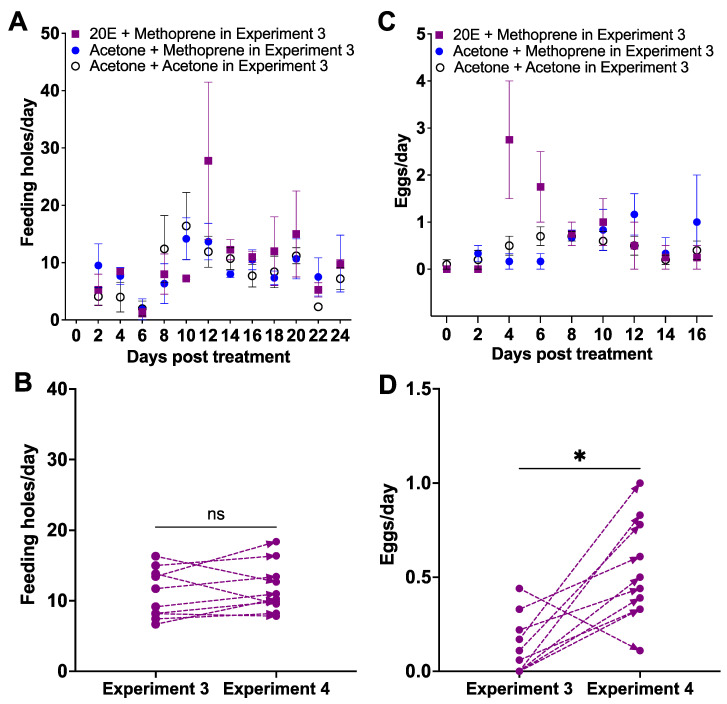
The effect of applying 1 μg of 20E followed by 1 μg of methoprene on underperforming (laying < 1 egg per day) and unresponsive females used in Experiment 3 (Figure 3). Daily mean number ± SE of feeding holes (**A**) and individual response of feeding holes for each female weevil (**B**) in Experiment 3 and treated with 20E and methoprene in Experiment 4; daily mean number of eggs ± SE (**C**) and individual response of eggs for each female weevil (**D**); ns: not significant; *: *p* < 0.05.

The feeding holes were similar among the three retreated groups from Day 8 to 24 (*Z* = 0.3751, *p* > 0.05, Figure 4A): the acetone + acetone group (Pre AA) (8.19 ± 1.30 feeding holes/day, mean ± SE), the acetone + methoprene group (Pre AM) (8.92 ± 0.96 feeding holes/day), and the 20E + methoprene group (Pre 2M) (10.27 ± 1.92 feeding holes/day). There was no difference in the number of feeding holes between underperforming and unresponsive females in Experiment 3 and their performance in Experiment 4 (paired Wilcoxon signed-rank test, *W* = 19, *p* = 0.3750; Figure 4B). Oviposition by the Pre AA group started on Day 8, versus Day 10 for the Pre AM group, and Day 12 for the Pre 2M group (Figure 4C). The egg production during the oviposition period from Day 8 to 24 was not different among three groups (*Z* = 0.28, *p* > 0.05; Figure 4C): the Pre AA group (0.43 ± 0.07 eggs/day), the Pre AM group (0.52 ± 0.14 eggs/day), and the Pre 2M group (0.81 ± 0.31 eggs/day, mean ± SE). All females treated with 1 μg of 20E and 1 μg of methoprene initiated oviposition regardless of their previous treatments (*W* = 46, *p* < 0.05; Figure 4D). The mortality was 16.7% for the Pre AA group, 40% for the Pre AM group and 0% for the Pre 2M group (χ^2^ = 52.37, df = 2, *p* < 0.0001): Pre 2M vs. Pre AM (χ^2^ = 50.00, df = 1, *p* < 0.0001), Pre 2M vs. Pre AA (χ^2^ = 18.58, df = 1, *p* < 0.0001), Pre AM vs. Pre AA (χ^2^ = 12.98, df = 1, *p* < 0.001).

## 4. Discussion

Our results demonstrated that doses of 0.01, 0.1 and 1.0 μg of methoprene progressively increased feeding by adult females. However, only the dose of 1 μg of methoprene stimulated oviposition. The fact that the egg production only occurred in 60% of females treated with 1 μg of methoprene in Experiment 1 suggests that 1 μg of methoprene is near the threshold for terminating diapause of female *C. basicorne* that had experienced only four weeks of cold. However, the dose–response curve suggests that increases in feeding rate occur slowly above about 0.4 μg, so it is possible that a dose lower than 1.0 may induce oviposition. Previous studies indicated that at least eight weeks of cold was required to terminate diapause of *C. basicorne* [49]. The dose–response of oviposition after the methoprene treatment was also reported in other insects. For example, topically applied 1 μg of methoprene dissolved in acetone induced 67% of female face flies, *Musca autumalis,* to become reproductively active vs. 38% for 0.5 μg [26]. One hundred % of female moths of *Caloptilia fraxinella* Ely (Lepidoptera:) developed vitellogenic oocytes after topical doses of 0.1 μg vs. 19% for 0.01 μg [38]. Experiment 2 demonstrated that 1 μg of methoprene could induce oviposition in 100% of females that previously failed to respond to 0.0, 0.01 or 0.1 μg of methoprene in Experiment 1. Females started ovipositing sooner, and produced more eggs per day in Experiment 2 (0.81 ± 0.28) than Experiment 1 (0.50 ± 0.17) (*T* = 3.38, df = 57, *p* < 0.01). A possible explanation for these differences could be the increased light intensity used in Experiment 2. Alternatively, these females were two weeks older and had been at warm temperature two weeks longer than in Experiment 1. In Experiment 2, feeding rate increased linearly during the preoviposition period (Days 1–7) and then gradually decreased during the first seven days of the oviposition period. A similar decrease in feeding rate was observed in naturally ovipositing females [21]. The artificially applied methoprene presumably acted like JH to stimulate egg maturation of diapausing females of *C. basicorne*, which enabled them to lay eggs [27,31,36,50].

We did not test 20E alone because it was previously demonstrated that 20E alone failed to terminate reproductive diapause of Colorado potato beetles [31], and we did not have enough insects available to test this treatment simultaneously with the other treatments. Treating 20E 2 days before methoprene increased both feeding holes and eggs compared to the control and the methoprene only treatments in Experiment 3, which shows that 20E further helped terminate reproductive diapause of female *C. basicorne*. Oviposition started on the same day for the AM and 2M treatments in Experiment 3, which suggests that the effect of 20E was to produce more eggs rather than reduce the time to initiate oviposition of *C. basicorne.* The higher egg production by the AM group compared with the AA group is consistent with the theory that exogenous JH treatment stimulates egg maturation; however, exogenous JH also inhibits CA activity and increases JH esterase activity, which degrades the exogenous JH [28,31]. Thus, application of methoprene alone is expected to produce only a temporary effect. However, 20E is thought to block activation of JH esterase, and it may also stimulate production of JH [23,31]. This is consistent with the result that the number of feeding holes and oviposition were higher for the 2M (20E + methoprene) treatment than the AM (methoprene) treatment in Experiment 3. Although 37.5% of females in the AA group became reproductively active without any hormone treatment, none of the eggs from the AA group was viable, indicating that the level of this activity was far below normal. Nevertheless, oviposition by some control females indicates that this cohort was approaching the end of diapause, which may have been a consequence of the long period of time held at cold dark conditions before testing.

The oviposition rate of females in Experiment 3 exposed to the AM treatment (0.1 μg of methoprene; 1.00 ± 0.13 eggs/day) was higher than in Experiments 1 (0.50 ± 0.17) and 2 (0.81 ± 0.28) (Exp. 3 vs. 1: *T* = 10.97, df = 80, *p* < 0.001; Exp. 3 vs. 2: *T* = 5.02, df = 19, *p* < 0.001). However, it is notable that in all three experiments, the oviposition rate was lower than that observed by non-diapausing females reported in [21]: (1.8 ± 1.2) (vs. Exp. 1: *T* = 3.43, df = 470, *p* < 0.001; vs. Exp. 2: *T* = 5.76, df = 509, *p* < 0.001; vs. Exp. 3: *T* = 5.65, df = 532, *p* < 0.001). Weevils were originated from Turkey, rather than Greece, and measured oviposition at 25/25 °C (12 h photoperiod) [21], rather than 21/17 °C (12 h photoperiod) in the current study. Although geographic origin may have had an effect, it is reasonable that the warmer temperature accounts for the higher oviposition rate that they observed, e.g., [51,52].

We conclude that 1 μg of methoprene alone was able to induce females to oviposit viable eggs, but that the combination of 20E (1 μg) followed by methoprene (1 μg) was the best treatment for artificially terminating reproductive diapause of female *C. basicorne*, because it increased oviposition rate by 51% above that of methoprene alone. Our findings can be used in two ways in mass-rearing biocontrol agents. First, to increase the number of biocontrol agents to release by artificially terminating reproductive diapause when necessary, which would permit producing more than one generation per year in the laboratory. Adults produced out of season could be stored at cold temperature (5 °C) until needed for release in the spring [53]. Second, by treating reproductively inactive females a week before field release at the time of year when they should be ovipositing, they will become reproductively active and attack the host plant. Therefore, this tool could both increase annual production of a univoltine agent in the laboratory and ensure that individuals being released are reproductively active, which should enhance establishment rate of univoltine biocontrol agents for invasive plants.

## Data Availability

A dataset was posted in Ag Data Commons at https://data.nal.usda.gov/, doi: 10.15482/USDA.ADC/1523115.
